# 

**Published:** 1877-05-20

**Authors:** 


					﻿Jzj
a
a
a
a
a
A
o
I—I
Q
a
3
a
p
o
fx
O
H
a
<1
a
£
O
Hj
cc
I—I
K
H
a
o
ce
3
—
a
o
a
a
H
Eh
a
a
a
Ph
a
CQ
<
a
a
Qi
VOL. VII.
MAY 20, 1877
No. 5.
THE
SOUTHERN MEDICAL RECORD.
A MONTHLY JOURNAL OF PRACTICAL MEDICINE.
QUJCQU1I) ER^SCIEIES ESTO BREVIS. ”
I
OD
w
o
a
o
o
g
g
d
3
i—i
n
a
i—i
o
*
<n
>
«
hi
fc.
a
a
i—i
a
>
p
i—t
a
w
PH
a>
OQ
O
P
i—I
a
A
A
tel
p
JAS. P. HARRISON & CO., Printers.
ATLANTA, GEORGIA.
TERMS: $2.00 per Annum in Advance—Club Rates, & Copies for $8.00.
				

